# Fast computation of genome-metagenome interaction effects

**DOI:** 10.1186/s13015-020-00173-2

**Published:** 2020-07-01

**Authors:** Florent Guinot, Marie Szafranski, Julien Chiquet, Anouk Zancarini, Christine Le Signor, Christophe Mougel, Christophe Ambroise

**Affiliations:** 1 Université Paris-Saclay, CNRS, Univ Évry, Laboratoire de Mathématiques et Modélisation d’Évry, 91037 Évry-Courcouronnes, France; 2 ENSIIE, 91037 Évry-Courcouronnes, France; 3 Université Paris-Saclay, AgroParisTech, INRAE, UMR MIA-Paris, 75005 Paris, France; 4 Plant Hormone Biology group, Biosystems Data Analysis group, Swammerdam Institute for Life Sciences, University of Amsterdam, Science Park 904, 1098 XH Amsterdam, The Netherlands; 5 Agroécologie, AgroSup Dijon, INRAE, Univ Bourgogne, Univ Bourgogne Franche-Comté, 21000 Dijon, France; 6 IGEPP, INRAE, Institut Agro, Univ Rennes, 35653 Le Rheu, France

**Keywords:** Statistical machine learning, Variable selection, Dimensionality reduction, Gene-environement interactions, GWAS, Genetic and metagenomic markers

## Abstract

**Motivation:**

Association studies have been widely used to search for associations between common genetic variants observations and a given phenotype. However, it is now generally accepted that genes and environment must be examined jointly when estimating phenotypic variance. In this work we consider two types of biological markers: genotypic markers, which characterize an observation in terms of inherited genetic information, and metagenomic marker which are related to the environment. Both types of markers are available in their millions and can be used to characterize any observation uniquely.

**Objective:**

Our focus is on detecting interactions between groups of genetic and metagenomic markers in order to gain a better understanding of the complex relationship between environment and genome in the expression of a given phenotype.

**Contributions:**

We propose a novel approach for efficiently detecting interactions between complementary datasets in a high-dimensional setting with a reduced computational cost. The method, named SICOMORE, reduces the dimension of the search space by selecting a subset of supervariables in the two complementary datasets. These supervariables are given by a weighted group structure defined on sets of variables at different scales. A Lasso selection is then applied on each type of supervariable to obtain a subset of potential interactions that will be explored via linear model testing.

**Results:**

We compare SICOMORE with other approaches in simulations, with varying sample sizes, noise, and numbers of true interactions. SICOMORE exhibits convincing results in terms of recall, as well as competitive performances with respect to running time. The method is also used to detect interaction between genomic markers in *Medicago truncatula* and metagenomic markers in its rhizosphere bacterial community.

**Software availability:**

An R package is available [4], along with its documentation and associated scripts, allowing the reader to reproduce the results presented in the paper.

## Introduction

Association studies are a popular approach for digging out genetic information relating to a given phenotype. To avoid confusion effects (e.g. stratification due to population origin) and improve the diagnostic, it is common practice to integrate environmental data in the analysis. These additional variables are generally few in number, of the order of tens.

In this paper we propose a generic method for taking thousands or even millions of environmental variables into consideration, with the aim of finding significant interactions between these variables and genetic markers. We illustrate the proposed algorithm on the genome of *Medicago truncatula (Fabaceae, Plantae)* and metagenomic markers in its rhizosphere bacterial community, but it could be applied in many other contexts.

### Gene-environment interactions

Genome-Wide Association Studies (GWAS) look for genetic markers linked to a phenotype of interest. Typically, hundreds of thousands of single nucleotide polymorphisms (SNPs) are analyzed with a limited sample size using high-density genotyping arrays. GWAS are a powerful tool for investigating the genetic architecture of complex biological processes and have been successful in identifying hundreds of associated variants. However, they have been able to explain only a small proportion of the phenotypic variations expected from classical linkage analyzes [46].

Some of the missing heritability may be uncovered by taking into account correlations among variables and epistasis [58, and references therein]. Another way to understand and improve the knowledge of complex phenotypes is to look at gene-environment interactions. If the contributions of genes and environment to a phenotype are examined separately and interactions between them ignored, this can give incorrect estimates of how much phenotypic variance is attributable to genes alone, to environment alone, and to genes and environment jointly.

Gene-environment interactions are clearly of great interest in medical genetics and epidemiology [15, 61] but also in plant research regarding environmental adaptation issues [30, 31]. In particular, Metagenome-Wide Association Analysis (MWAS) [55, 65, 66] is providing a growing body of evidence regarding the role of gut microbiome in basic biological processes and in the development and progression of major human diseases, such as infectious diseases, gastrointestinal cancers, and metabolic diseases. In plants, the role of the rhizosphere^1^ microbiome on the plant growth and health is well known and has been studied since the early 2000s [8, 45, 49, 52]. While GWAS analyses have been able to identify associations between the plant genome of *Arabidopsis thaliana* and the metagenome (amplicon sequencing) of its associated phyllosphere and root microbial communities [9, 34], in plants, to our knowledge, no specific MWAS analyses have so far been done.

### Combining genome and metagenome analyses

There have been a number of works regarding the integration of multi-omics data in statistical or machine learning models, with several review papers. For instance, Li et al. [41] establish a typology regarding different families of models. Huang et al. [35] also list the kind of omics data which can be used and the outputs given by the methods. Hawe et al. [32] pay attention to the inference of interaction networks.

However, these methods do not include environmental variables and consequently fail to address specificities of such features. There exists literature discussing both microbiome and genetics. They are mainly classical methods applied to a reduced set of species-gene pairs [38]. Another way of relating genetic and metagenomic data is to consider the metagenome as a phenotype and to perform quantitative trait locus (QTL) mapping. This kind of metagenomic QTL analysis illustrates the role of host genetics in shaping metagenomic diversity between individuals [57, 67].

An alternative of interest is to consider metagenomic variables as environmental variables in GWAS. Several quantitative approaches have been proposed in classical gene-environment interaction studies with a small number of environmental factors limited to certain modalities, such as different status (smoking / non smoking, for instance) or medical treatments [29, 36]. More specifically, our proposal shares similarities with approaches where interactions can be modelled using a classical (generalized) linear model with interaction terms [44].

However, the number of interactions that need to be tested may increase dramatically when metagenomic markers are considered as environmental data. In this perspective, variable selection or variable compression may be of use here as a means of reducing the dimension of the problem in order to design an efficient method for detecting gene-environment interaction in a high-dimensional setting.

### Taking structures into account in association studies

Data compression for dimension reduction may be achieved in various ways. A distinction is usually drawn between feature selection and feature extraction. Feature selection consists in selecting a few relevant variables from among the original variables, whereas feature extraction consists in computing new representative variables.

For the kind of association study that concerns us here, feature selection is often preferred to feature extraction for interpretative purposes. In this paper we advocate a mixed approach including feature extraction that is based on the underlying structures of the genome and the metagenome, combined with feature selection.

The idea of considering group structures is not new. It has already been advocated both in the context of GWAS [17] and MWAS [53]. In the context of prediction from gene expression regression, Park et al. [50] proposed clustering genes hierarchically to obtain a dendrogram that reveals their nested correlation structure. At each level of the hierarchy, supergenes are computed as the average expression of the current clusters. It can be shown that regressing over supergenes improves precision if the correlation structure is sufficiently strong. In a similar fashion, Guinot et al. [27] made use of the haplotype structure of the human genome when they proposed a dimension-reduction approach that can be applied in the context of GWAS. It is worth noting that similar ideas have also been developed in other areas such medical imaging [14].

### Contributions and organization of the paper

In this work, we propose a method for detecting interactions between genomic and metagenomic data. The method comprises four steps. Given a dataset:

1. Identify a group structure within the variables using a hierarchical clustering;
2. Create compressed features, or *supervariables*, according to this group structure;
3. Select a subset of supervariables using a Lasso procedure with a penalty factor weighted by the length of the gap between two successive levels of a hierarchical clustering;
4. Combine the two compressed datasets in a linear model with interactions in order to perform multiple hypothesis testing.

This scheme allows interactions to be detected efficiently in a high-dimensional setting with a reduced computational cost.

The paper is organized as follows. "Learning interactions with complementary datasets" section looks at the role of linear models of interactions and proposes a framework for learning using complementary datasets. "Method" section describes our method, which seeks to uncover relevant interactions using, first, compressions of data based on hierarchical structures, second, a Lasso selection procedure and, third, model testing. Finally, "Numerical simulations" section provides an illustration of our approach using numerical simulations, and "Application on the rhizosphere bacterial communities of *Medicago truncatula*" section describes an application for examining interactions between the genomic markers of the species *Medicago truncatula* and metagenomic markers of its rhizosphere microbial community.

## Learning interactions with complementary datasets

This section gives a general introduction together with some notation, and outlines how we will establish a compact model of interactions between complementary datasets.

### Remark 1

Here, and in what follows, the term *genomic* data will refer to SNP data. In "Learning interactions with complementary datasets", "Method" and "Numerical simulations" sections, we will use the term *metagenomic* data for metabarcoding or shotgun data. The application on *Medicago truncatula* will be described in greater detail. Extensions to other kinds of data will be discussed in Section Conclusion.

### Setting and notations

Let us consider observations from two complementary views, $G$ (for Genomic data) and $M$ (for Metagenomic data), which are placed together in a training set $\mathcal {S} = \{(\mathbf {x}^ G _i, \mathbf {x}^ M _i, y_i)\}_{i=1}^N$, where $(\mathbf {x}^ G _i, \mathbf {x}^ M _i, y_i) \in \mathbb {R}^{D_ G } \times \mathbb {R}^{D_ M } \times \mathbb {R}$.

We assume the existence of underlying biological information on $G$ and $M$, encoded as groups. The group structure over $G$ is defined by $N_{ G }$ groups of variables $\mathcal {G}=\{\mathcal {G}_{g} \}_{g=1}^{N_{ G }}$. We denote as $\mathbf {x}_i^{g} \in \mathbb {R}^{D_{g}}$ the sample *i* restricted to the variables of $G$ from group $\mathcal {G}_{g}$. Similarly, the group structure over $M$ is defined by $N_{ M }$ groups of variables $\mathcal {M}=\{\mathcal {M}_{m}\}_{m=1}^{N_{ M }}$, and $\mathbf {x}_i^{m} \in \mathbb {R}^{D_{m}}$ is the sample *i* restricted to the variables of $M$ from group $\mathcal {M}_{m}$.

We also introduce $D_I = D_{ G } \cdot D_{ M }$ and $N_I = N_{ G } \cdot N_{ M }$, corresponding to the number of variables and the number of groups that may interact.

Finally, we use the following convention: vectors of observations indexed with *i*, such as $\mathbf {x}_i$, will usually be row vectors, while vectors of coefficients, such as $\varvec{\beta }$, will usually be column vectors.

### Interactions in linear models

Interactions between data from views $G$ and $M$ may be captured in the model

1 $$\begin{aligned} y_i = \mathbf {x}^{ G }_i \varvec{\gamma }_{ G } + \mathbf {x}^{ M }_i \varvec{\gamma }_{ M } + \mathbf {x}^{ G }_i \varvec{\Delta }_{ G M } (\mathbf {x}^{ M }_i)^T + \epsilon _i \,, \end{aligned}$$

where the vectors $\varvec{\gamma }_{ G } \in \mathbb {R}^{D_{ G }}$ and $\varvec{\gamma }_{ M } \in \mathbb {R}^{D_{ M }}$ denote the linear effects related to $G$ and $M$ respectively, the matrix $\varvec{\Delta }_{ G M } \in \mathbb {R}^{D_{ G } \times D_{ M }}$ contains the interactions between all pairs of variables in $G$ and $M$, and $\epsilon _i \in \mathbb {R}$ is a residual error.

Models with interactions distinguish between *strong dependency* (SD) and *weak dependency* (WD). *Strong dependency* is the more common hypothesis (see for instance [10] and the discussion therein), and it means that an interaction is effective if and only if the corresponding single effects are also effective. *Weak dependency*, on the other hand, means that an interaction is effective if one of the main effects is also effective. Formally, for all variables $j \in \mathbf {x}^{ G }$ and for all variables $j' \in \mathbf {x}^{ M }$, if $\gamma _{j}$, $\gamma _{j'}$ and $\delta _{jj'}$ are the coefficients related to $\varvec{\gamma }_{ G }$, $\varvec{\gamma }_{ M }$ and $\varvec{\Delta }_{ G M }$, then

$$\begin{aligned}&(SD)&\delta _{jj'} \ne 0 \qquad \Rightarrow \qquad \gamma _{j} \ne 0&\text { and }&\gamma _{j'} \ne 0 \,, \\&(WD)&\delta _{jj'} \ne 0 \qquad \Rightarrow \qquad \gamma _{j} \ne 0&\text { or }&\gamma _{j'} \ne 0 \,. \end{aligned}$$

In this context, Bien et al. [10] proposed a sparse model of interactions that is likely to encounter computational limitations for large-dimensional problems ([42] and [56]). Lim [42] present a method for learning pairwise interactions in a regression model by solving a constrained overlapping group Lasso [37] in a manner that satisfies strong dependencies. She et al. [56] propose a formulation with an overlapping regularization that fits both types of hypothesis, and they provide theoretical insights on the resulting estimators. 2

However, the dimension $D_{ G } + D_{ M } + D_I$ inherent in Problem (1) when estimating $\varvec{\gamma }_{ G }$, $\varvec{\gamma }_{ M }$ and $\varvec{\Delta }_{ G M }$ may be inconveniently large, especially for applications with numerous variables such as in biology with genomic and metagenomic markers. To reduce this dimension we propose compressing the data according to an underlying structure that may be defined on the basis of prior knowledge or uncovered using clustering algorithms.

### Compact model

Let us consider that if we have a compression function for all groups $G$ and $M$, we can shape Problem (1) into a compact form

2 $$\begin{aligned} y_i = \sum _{g\in \mathcal {G}} \tilde{x}_i^g\beta _{g} + \sum _{m\in \mathcal {M}} \tilde{x}_i^m\beta _{m} + \sum _{g\in \mathcal {G}} \sum _{m\in \mathcal {M}} \underbrace{\left( \tilde{x}_i^g\cdot \tilde{x}_i^m\right) }_{\phi ^{gm}_i} {\theta _{gm}} + \, \epsilon _i \,, \end{aligned}$$

where $\tilde{x}_i^g\in \mathbb {R}$ is the $i^{th}$ compressed sample of the variables that belong to the group $g$ for the view $G$, and $\beta _{g} \in \mathbb {R}$ is its corresponding coefficient. The counterparts in the group $m$ for the view $M$ are $\tilde{x}_i^m\in \mathbb {R}$ and $\beta _{m} \in \mathbb {R}$. Finally, $\theta _{gm} \in \mathbb {R}$ is the interaction between groups $g$ and $m$.

Problem (2) can be reformulated in a vector form. Let $\tilde{\mathbf {x}}_{i} \in \mathbb {R}^{N_{ G }}$, $\varvec{\beta }_{ G } \in \mathbb {R}^{N_{ G }}$, $\tilde{\mathbf {x}}_{i} \in \mathbb {R}^{N_{ M }}$ and $\varvec{\beta }_{ M } \in \mathbb {R}^{N_{ M }}$ be

$$\begin{aligned} \tilde{\mathbf {x}}_{i}^{ G }&= (\tilde{x}_i^1 \cdots \tilde{x}_i^g\cdots \tilde{x}_i^{N_{ G }})\,,&\varvec{\beta }_{ G }&= (\beta _{1} \cdots \beta _{g} \cdots \beta _{N_{ G }})^{T}\,,\\ \tilde{\mathbf {x}}_{i}^{ M }&= (\tilde{x}_i^1 \cdots \tilde{x}_i^m\cdots \tilde{x}_i^{N_{ M }}) \,,&\varvec{\beta }_{ M }&= (\beta _{1} \cdots \beta _{m} \cdots \beta _{N_{ M }})^{T}\,. \end{aligned}$$

We denote as $\varvec{\phi }_{i} \in \mathbb {R}^{N_I}$ the vector whose general component is given by $\phi ^{gm}_i$ in Equation (2), that is

$$\begin{aligned} \varvec{\phi }_{i}&= \left( \phi ^{11}_i \cdots \phi ^{1N_{ M }}_i \cdots \phi ^{gm}_i \cdots \phi ^{{N_{ G }} 1}_i \cdots \phi ^{{N_{ G }} {N_{ M }}}_i \right) \,, \end{aligned}$$

and $\varvec{\theta }\in \mathbb {R}^{N_I}$ denotes the corresponding vector of coefficients, that is

$$\begin{aligned} \varvec{\theta }=&\left( \theta _{11} \cdots \theta _{1 {N_{ M }}} \cdots \theta _{gm} \cdots \theta _{{N_{ G }} 1} \cdots \theta _{{N_{ G }} {N_{ M }}} \right) ^T\,. \end{aligned}$$

Finally, Problem (2) reads as a classical linear regression problem

3 $$\begin{aligned} y_i = \tilde{\mathbf {x}}_{i}^{ G } \varvec{\beta }_{ G } + \tilde{\mathbf {x}}_{i}^{ M } \varvec{\beta }_{ M } + \varvec{\phi }_{i} \varvec{\theta }+ \epsilon _i \,, \end{aligned}$$

øof dimension $$N_{ G } + N_{ M } + N_I$$.

### Uncovering relevant interactions

Compared to Problem (1) and provided that $$N_ G $$ and $$N_ M $$ are reasonably smaller than $$D_{ G }$$ and $$D_{ M }$$, the dimension of Problem (3) is drastically reduced, so that it may be solved with the aid of a suitable optimization algorithm and sufficient computing resources. For instance, Donoho and Tsaig [18] give an overview of $$\ell _1$$ regularized algorithms to solve sparse problems like Lasso, which in our case could take the form:

$$\begin{aligned} \left\{ \begin{array}{ll} \mathop {\mathrm{arg min}}\limits _{\varvec{\beta }_{ G },\, \varvec{\beta }_{ M },\, \varvec{\theta }} &{} \quad \sum _{i=1}^n \left( y_i - \tilde{\mathbf {x}}_{i}^{ G } \varvec{\beta }_{ G } - \tilde{\mathbf {x}}_{i}^{ M } \varvec{\beta }_{ M } - \varvec{\phi }_{i} \varvec{\theta }\right) ^2 \\ \\ &{} \quad + \lambda _ G \sum _{g=1}^{N_{ G }} |\beta _{g}| + \lambda _ M \sum _{m=1}^{N_{ M }} |\beta _{m}| + \lambda _I \sum _{g,m=1}^{N_I} |\theta _{gm}| \,, \end{array}\right. \end{aligned}$$

with $\lambda _ G$, $\lambda _ M$ and $\lambda _I$ being the positive hyperparameters that respectively control the amount of sparsity related to coefficients $\varvec{\beta }_ G$, $\varvec{\beta }_ M$ and $\varvec{\theta }$. The $N_ G + N_ M + N_I$ dimension may nevertheless remain large in relation to the number of observations *N*. Also, it will be remarked that this kind of formulation does not automatically entail the dependency hypotheses (SD) and (WD) unless additional constraints are introduced. For this purpose, the works by Bien et al. [10], Lim and Hastie [42] or She et al. [56] mentioned above may be considered. In the following section we present another way of reducing the dimension further and ensuring that the strong dependency hypothesis is satisfied.

## Method

In this section we provide some elements for addressing Problem (3) in relation to biological problems involving complementary datasets. Our proposed approach, which we have named SICOMORE (Selection of Interaction effects in COmpressed Multiple Omics REpresentations), is available for download as an R package [4].

### Preprocessing of the data

When tackling problems that involve genomic and metagenomic interactions, some prior transformations are necessary. This preliminary step may also include a first attempt at reducing the dimension.

#### Transformation for metagenomic data

Metagenome sequencing gives rise to features that take the form of proportions in different samples. This kind of information is referred to in the statistical literature as compositional data [2] and is known to be subject to negative correlation bias [2, 51]. The most common way to circumvent this issue is to transform the $D_{ M }$ features using centered log-ratios and to replace 0 values using maximum-likelihood approaches (see [20, 21] and references therein). A more detailed presentation of these aspects may be found in [54].

#### Initial selection of variables

As described in "Learning interactions with complementary datasets" section, we make the assumption that interactions have strong dependencies, which means that an interaction can be effective only if the two simple effects associated with the variables in interaction are included in the model. For this reason it may be advantageous to make an initial selection in order to eliminate inoperative single effects on $G$ and $M$ respectively. Different approaches for carrying out this selection may be considered. For example, screening rules can eliminate variables that will not contribute to the optimal solution of a sparse problem, sweeping all the variables upstream to the optimization. In cases where this kind of screening is appropriate, the work of Lee et al. [39] is a useful resource. Their focus is on Lasso problems and they present an overview of these techniques, together with an ensemble of screening rules. Once the screening has been performed, the optimization of a Lasso problem gives the final set of variables.

### Structuring the data

Once the data have been preprocessed, hierarchical clustering using Ward’s method with appropriate distances can be employed to uncover the tree structures.

#### Clustering of metagenomic data

Several approaches are available for analyzing microbiota compositions. Li [40] has produced a review of statistical and computational methods according to different objectives and/or technologies. For problems with numerous similar reference sequences, Fischer et al. [19] have proposed a general linear model approach designed to estimate taxon abundances for strain-level analyses.

A commonly used approach when analyzing metabarcoding data is to group sequences into taxonomic units [11]. The features arising from such a sequencing are often modeled as Operational Taxonomic Units (OTUs), each OTU representing species proxies according to some degree of sequence similarity. More recent methods based on denoising techniques have led to the definition of Amplicon Sequence Variants (ASVs), which can be considered as refined versions of OTUs [13].

While the structure of microbial communities can be defined according to the underlying phylogenetic tree, it also makes sense to use more classical distances to define a hierarchy based on the abundance of OTUs. In our application, we use an agglomerative hierarchical clustering with the Ward criterion.

#### Clustering of genomic data

When the genomic information is available through SNP, the tree structure on $G$ will be defined using a hierarchical clustering algorithm that integrates the linkage disequilibrium as the measure of dissimilarity [17].

This algorithm is a computationally efficient hierarchical clustering that makes use of the structure of the genome in order to cluster SNPs into adjacent groups. More specifically, it is a spatially constrained hierarchical clustering based on Ward’s incremental sum-of-squares algorithm [68] in which the measure of dissimilarity is based on the linkage disequilibrium between SNPs *j* and $j'$: $1 - r^2(j,j')$. The algorithm also makes use of the fact that the linkage disequilibrium matrix can be modeled as group-diagonal by allowing only groups of variables that are adjacent on the genome to be merged, which significantly reduces the computational cost.

### Using the structure efficiently

Different approaches for finding an optimal number of clusters may be envisaged when looking for the optimal cut in a tree structure obtained by hierarchical clustering (see for instance [48] or [23]). Whatever the approach, finding this optimal cut necessarily involves a systematic exploration of different levels of the hierarchy. Our alternative strategy for bypassing this expensive exploration is as follows:

- Expanding the hierarchy, considering all possible groups at a single level;
- Assigning a weight to each group based on the distances between two consecutive groups in the hierarchy;
- Compressing each group into a supervariable.

**Fig. 1:**
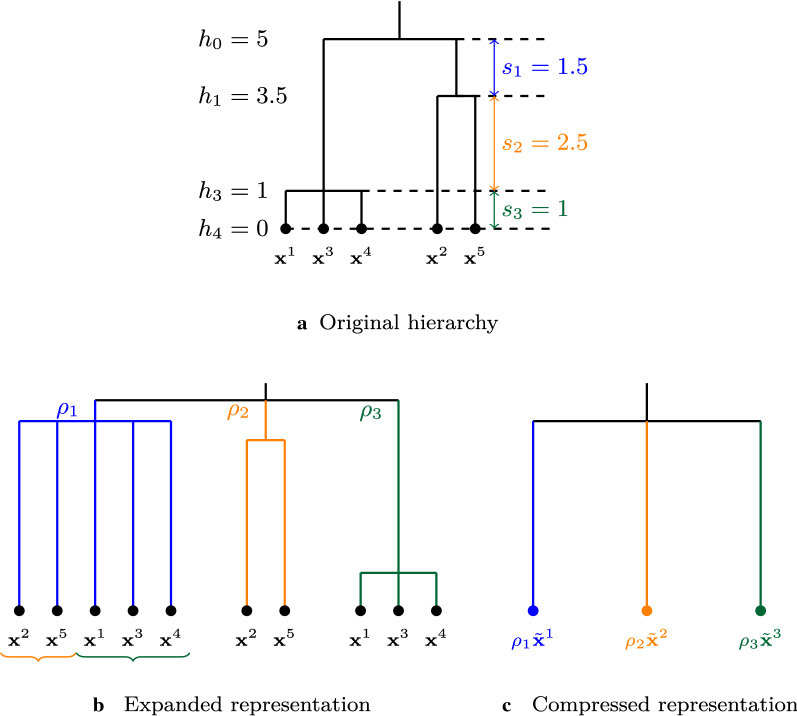
Dimension reduction strategy. **a** Original hierarchical tree with an example for 5 variables. **b** Expanded representation of the tree with all possible weighted groups derived from the original hierarchy. The group in blue gathers the variables contained in the groups in orange and green. **c** Compressed representation of the tree after construction of the supervariables

The different steps in this strategy are illustrated in Fig. 1, from the original tree structure in Fig. 1a to the final flattened, weighted, compressed representation shown in Fig. 1c.

#### Expanding the hierarchy

To reduce the dimension of Problem (3), the first step consists in flattening the respective tree structures obtained on views $G$ and $M$ so that only one group structure remains. Each group of variables defined at the deepest level may thus be included in other groups of larger scales, as shown in Fig. 1b.

#### Assigning weights to the groups

To keep track of the tree structure, an additional measure may be included to quantify the loss of information between two successive levels. More specifically, for a tree structure of height *H* and for $1 \le h \le H-1$, we define $s_h$ as the gap between heights *h* and $h-1$. Using a similar methodology to Grimonprez [25] for the multi-layer group Lasso, we define this quantity as $\displaystyle {\rho _h = 1 / \sqrt{s_h}}$. The process is shown in Fig. 1a and 1b.

#### Compressing the data

To summarize each group of variables the mean, the median, or other quantiles may be used, as well as more sophisticated representations based on eigenvalue decomposition, such as the first factor of a Principal Component Analysis.

### Identification of relevant supervariables

With the aid of this compressed representation we can uncover relevant interactions using a multiple testing strategy.

#### Selection of supervariables

Compression is a key ingredient in reducing significantly the dimension of Problem (3). We take this a step further with an additional feature selection process applied to the compressed variables, as described at the beginning of this section, in order to preprocess the data using screening rules and/or applying a Lasso optimization on each view $G$ and $M$:

$$\begin{aligned} \mathop {\mathrm{arg min}}\limits _{\varvec{\beta }_{ G }}&\; \sum _{i=1}^n \left( y_i - \tilde{\mathbf {x}}_{i}^{ G } \varvec{\beta }_{ G } \right) ^2 \ + \ \lambda _ G \sum _{g=1}^{N_{ G }} \rho _g|\beta _{g}| \,, \end{aligned}$$

and

$$\begin{aligned} \mathop {\mathrm{arg min}}\limits _{\varvec{\beta }_{ M }}&\; \sum _{i=1}^n \left( y_i - \tilde{\mathbf {x}}_{i}^{ M } \varvec{\beta }_{ M } \right) ^2 \ + \ \lambda _ M \sum _{m=1}^{N_{ M }} \rho _m|\beta _{m}| \,, \end{aligned}$$

with penalty factors defined by $\displaystyle {\rho _g= 1 / \sqrt{s_g}}$ and $\displaystyle {\rho _m= 1 / \sqrt{s_m}}$, as explained in "Structuring the data" section.

This step for selecting the supervariables in the two complementary datasets can be subject to instability when setting the amount of selection. The method can be improved further in terms of model consistency by using resampling techniques [5, 33, 47]. This has been implemented in SICOMORE with the R package stabs [6].

#### Linear model testing

For the purpose of feature selection the relevant interactions may be uncovered separately by considering each selected group $g\in \mathcal {G}$ coupled with each selected group $m\in \mathcal {M}$ in a linear model of interaction and by performing a hypothesis test (a standard *t*-test for instance) on each parameter ${\theta _{gm}}$:

4 $$\begin{aligned} y_i = \tilde{x}_i^g\beta _{g} + \tilde{x}_i^m\beta _{m} + \left( \tilde{x}_i^g\cdot \tilde{x}_i^m\right) {\theta _{gm}} + \, \epsilon _i \,. \end{aligned}$$

This strategy has the advantage of highlighting all the potential interactions between the selected simple effects in an exploratory rather than a predictive analysis perspective. It can also be seen as an alternative way of shortcutting Problem (3), in that it involves $N_I$ problems of dimension 3 rather than a potentially large problem of dimension $N_{ G } + N_{ M } + N_I$. Finally, by construction, this selection scheme preserves strong dependencies.

## Numerical simulations

We present some numerical simulations to assess SICOMORE’s ability to uncover relevant interactions. We compare our approach with two other methods, namely MLGL [25] and glinternet [42]. These two methods will be described in more detail later in the section. Both are available as R packages on the CRAN platform [26, 43].

These numerical simulations are designed to study several aspects of SICOMORE:

- The ability to recover relevant interactions will be observed on different configurations with respect to the sample sizes, the noise, and the number of true interactions.
- The impact of the weighting scheme will be shown with two versions of our approach, using both weighted and unweighted supervariables.
- The impact of the compression scheme will be compared to MLGL using the same structure but with the initial variables.
- Finally, a dedicated simulation sketches the running times necessary for each method to reach convergence when the dimension of one of the matrices grows. To allow the comparison of SICOMORE with MLGL or glinternet, the dimensions of the simulated matrices have been kept between a few hundred and a few thousand.

### Data generation

#### Generation of metagenomic and genomic data matrices

**Fig. 2:**
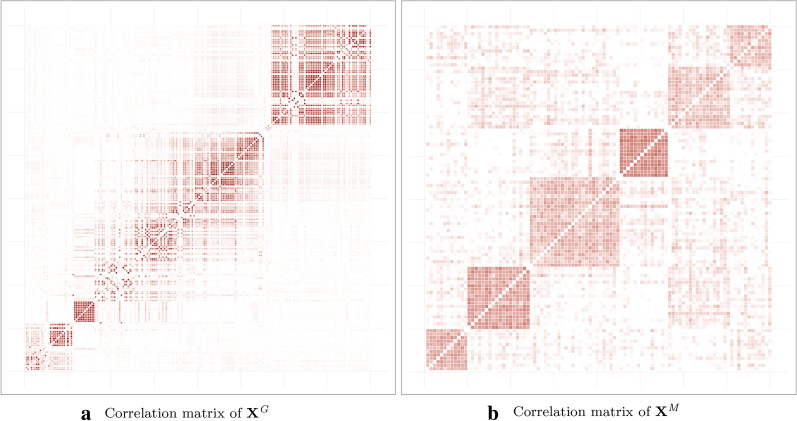
Examples of group structures: correlations observed on (**a**) genomic data $\mathbf {X}^ G$ and **b** metagenomic data $\mathbf {X}^ M$

*Genomic data.* To obtain a matrix $\mathbf {X}^{ G }$ resembling real genomic data we used HAPGEN2 software  [59, 60], which can simulate an entire chromosome conditionally on a reference set of population haplotypes (from HapMap3) and an estimate of the fine-scale recombination rate across the region, so that the simulated data share similar patterns with the reference data. We generated chromosome 1 using the haplotype structure of CEU population (Utah residents with Northern and Western European ancestry from the CEPH^3^ collection) as the reference set, and we selected $D_ G =200$ variables from this matrix to obtain the simulated dataset. An example of the linkage disequilibrium structure among the simulated SNPs is shown in Fig. 2a.

*Metagenomic data.* The data matrix $\mathbf {X}^{ M }$, with $D_ M =100$ variables, was generated using a multivariate Poisson log-normal distribution [1] with group structure dependencies. The Poisson log-normal model is a latent Gaussian model where latent vectors $\mathrm {Z}_i \in \mathbb {R}^{D_ M }$ are drawn from a multivariate normal distribution

$$\begin{aligned} \mathrm {Z}_i \sim \mathcal {N}_{D_ M }(0, \varvec{\Sigma }) \ , \end{aligned}$$

and where $\varvec{\Sigma }$ is a covariance matrix that can give a correlation structure between the variables. The random variable $\mathrm {X}^{ M }_i$ related to the centered phenotypic count data is then drawn from a Poisson distribution conditionally on $\mathrm {Z}_i$

$$\begin{aligned} \mathrm {X}^{ M }_{ij}|\mathrm {Z}_{ij} \sim \mathcal {P}\left( e^{\mu _j + \mathrm {Z}_{ij}}\right) . \end{aligned}$$

The group structure shown in Fig. 2b was obtained by drawing a latent multivariate normal vector using a covariance matrix such that the correlation level between the latent variables in a group are between 0.5 and 0.95. Simulating in this way gives a matrix of count data with a covariance structure close to what is observed with metagenomic data. As described in Section 3.1, we computed the proportions for each of the random variables and transformed them using centered log-ratios.

#### Generation of the phenotype

For all simulations we used a fixed value of $N_{ M } = 6$ groups for the matrix $\mathbf {X}^{ M }$. For the matrix $\mathbf {X}^{ G }$, since HAPGEN2 does not allow the group structure to be controlled exactly, we used the gap statistic [62] to identify a number of groups in the hierarchy. For instance, in Fig. 2a, the gap statistic identified $N_{ G } = 16$ groups. The supervariables were then calculated using averaged groups of variables to obtain the two matrices of supervariables, $\tilde{\mathbf {X}}^{ G }$ and $\tilde{\mathbf {X}}^{ M }$.

To generate the phenotype, we considered a data structure for which the data to regress was generated using supervariables according a linear model with interactions of the form:

5 $$\begin{aligned} y_i = \sum _{g\in \mathcal {S}^{ G }}\tilde{x}_i^g\beta _{g} + \sum _{m\in \mathcal {S}^{ M }} \tilde{x}_i^m\beta _{m} + \sum _{g\in \mathcal {S}^{ G }}\sum _{m\in \mathcal {S}^{ M }} \underbrace{\left( \tilde{x}_i^g\cdot \tilde{x}_i^m\right) }_{\phi ^{gm}_i} {\theta _{gm}} + \, \epsilon _i \,, \end{aligned}$$

where $\mathcal {S}^{ G }$ and $\mathcal {S}^{ M }$ are subsets of randomly chosen effects from the matrices $\tilde{\mathbf {X}}^{ G }$ and $\tilde{\mathbf {X}}^{ M }$ respectively, $\tilde{x}_i^g$ is the $i^{th}$ sample of the $g$ effect and $\beta _{g}$ its corresponding coefficient, and $\tilde{x}_i^m$ is the $i^{th}$ sample of the $m$ effect and $\beta _{m}$ its corresponding coefficient. Finally, $\theta _{gm}$ is the interaction between variables $\tilde{x}_i^g$ and $\tilde{x}_i^m$.

We considered $I \in \{1,3,5,7,10\}$ true interactions between some supervariables to generate the phenotype such that *I* blocks of the coefficients of $\theta _{gm}$ have non zero values. The process was repeated 30 times for each couple of parameters in $N=\{50, 100, 200\} \times sd(\varvec{\epsilon })=\{0.5, 1, 2\}$.

### Comparison of methods

In accordance with the outline given in the preamble of Section 4, we were seeking to assess the ability of SICOMORE, in comparison with MLGL and glinternet, to uncover true causal interactions. For this purpose, we needed to reshape the datasets provided to the two methods as we now describe below.

It is worth mentioning that SICOMORE is an approach that draws on the work of Park et al. [50] and MLGL [25], with an explicit design for detecting interactions. We explore two settings : $\rho$-SICOMORE and SICOMORE, which correspond respectively to the method described in section 3 using $\displaystyle {\rho _h = 1 / \sqrt{s_h}}$ and $\rho _h = 1$, $\forall h$.

#### Multi-Layer Group Lasso (MLGL)

Grimonprez [25] defines MLGL as a two-step procedure that combines a hierarchical clustering with a group Lasso regression. It is a weighted version of the overlapping group Lasso [37] which performs variable selection on multiple group partitions defined by the hierarchical clustering. A weight is attributed to each possible group identified at all levels of the hierarchy, as described in "Assigning weights to the groups" section. This weighting scheme favors the creation of groups associated with large gaps in the hierarchy.

The model of interactions is fitted with weights on the groups defined by the expanded representation of the two hierarchies using the initial variables, as illustrated in Fig.1b. The ability of MLGL to uncover real interactions is evaluated positively if it selects the correct interaction terms between two groups of variables at the right level in both hierarchies.

It should be noted that here MLGL is not being evaluated in a context for which it was intended, since MLGL examines the different levels of a hierarchical structure using *all* variables. This approach is not well suited in a high-dimensional setting and still less in a model of interactions. But, as we explained at the beginning of "Numerical simulations" section, this comparison with MLGL is intended to shed light on the impact of the compression applied to the variables in SICOMORE.

#### Group Lasso interaction network (glinternet)

Lim and Hastie [42] introduced glinternet, a procedure that considers pairwise interactions in a linear model in a way that satisfies strong dependencies between main and interaction effects: whenever an interaction is estimated to be non-zero, its two corresponding main effects are also included in the model.

It fits a hierarchical group Lasso model, with constraints on the main and interactions effects, as specified in "Uncovering relevant interactions" section, and it accommodates the strong dependency hypothesis by adding an appropriate penalty to the loss function (we refer the reader to [42] for more details on the form of the penalty). For very large problems (with a number of variables $\ge 10^5$), the group Lasso procedure is preceded by a screening step that gives a candidate set of main effects and interactions.

Since this method can only work at the level of variables, we needed to include a group structure into the analysis, and so we decided to fit the glinternet model on the compressed variables and to constrain the model to only fit the interaction terms between the supervariables of the two matrices $\tilde{\mathbf {X}}^{ G }$ and $\tilde{\mathbf {X}}^{ M }$. We explicitly removed all interaction terms between supervariables belonging to the same data matrix.

To ensure that our comparison of SICOMORE was fair, we considered two options, namely GLtree and GLgap. The GLtree option works on the unweighted compressed representations of the two hierarchies (Fig. 1c) and thus takes into account all the possible interactions between the supervariables of the two datasets. In contrast, the GLgap option considers only the interactions between the compressed variables constructed at a specific level in the hierarchies, chosen by the gap statistic. Given that $D^{ G }$ and $D^{ M }$ are the numbers of variables in $\mathbf {X}^{ G }$ and $\mathbf {X}^{ M }$, the dimension of the matrices $\tilde{\mathbf {X}}^{ G }$ and $\tilde{\mathbf {X}}^{ M }$ in GLtree are respectively $\tilde{D}^{ G } = D^{ G } + (D^{ G }-1)$ and $\tilde{D}^{ M } = D^{ M } + (D^{ M }-1)$.^4^ Consequently, for GLtree the number of interactions to be examined is $\tilde{D}^{ G } \times \tilde{D}^{ M }$, while for GLgap this number will depend on the level chosen by the gap statistic, but it will necessarily be smaller since this option considers only a specific level of the hierarchy. In the numerical simulations, given that $D^{ G } = 200$ and $D^{ M }=100$, the use of strong rules to discard variables is therefore not necessary.

### Evaluation metrics

For each run we evaluated the quality of the variable selection using Precision and Recall. More precisely, we compared the true interaction matrix ${\varvec{\theta }}$ that we used to generate the phenotype with the estimated interaction matrix $\hat{\varvec{\theta }}$ computed for each model.

For all possible $D_{ G } \times D_{ M }$ interactions, with $\theta _{jj'}$ the interaction term between variable $j \in \mathbf {X}^{ G }$ and variable $j' \in \mathbf {X}^{ M }$, we determined the following confusion matrix:

$$\begin{aligned} \begin{array}{c|c|c} &{} \hat{\theta }_{jj'}=0 &{} \hat{\theta }_{jj'}\ne 0 \\ \hline {\theta }_{jj'}=0 &{} \hbox { True Negative } &{} \hbox { False Positive }\\ \hline {\theta }_{jj'}\ne 0 &{} \hbox { False Negative } &{} \hbox { True Positive }\\ \end{array} \end{aligned}$$

**Fig. 3:**
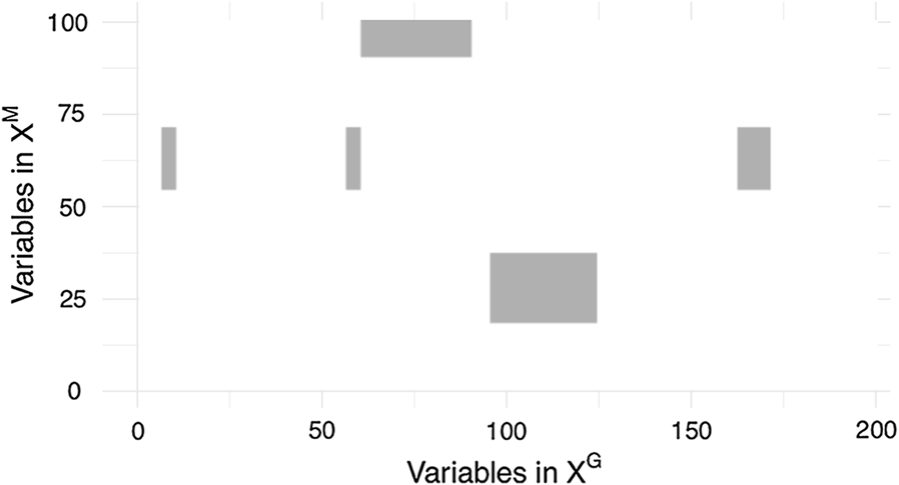
Illustration of the true block interaction matrix $\varvec{\theta }$ with $I = 5$, $\sigma = 0.5$ and $n=100$. Each non-zero value in this matrix is considered as a true interaction between two variables

The performances are measured with $\text {Precision}={\frac{TP}{FP+TP}}$ and $\text {Recall}=\frac{TP}{FN+TP}$. An example of the interaction matrix $\hat{\varvec{\theta }}$ is shown in Fig. 3 for $I=5$ blocks in interaction.

Here, a *true positive* corresponds to a significant *p*-value on a true causal interaction, a *false positive* to a significant *p*-value on a noise interaction, and a *false negative* to a non-significant *p*-value on a true causal interaction.

For the three tested methods we corrected for multiple testing by controlling the family-wise error rate with the Holm-Bonferroni method. Even though it is known to be stringent, we chose the Holm-Bonferroni method to adjust for multiple testing because the number of hypothesis tests that needed to be performed for our simulation was quite low. In a high-dimensional context, for example in analyzing real microarray data, the Benjamini-Hochberg method would be preferable for controlling the false discovery rate.

### Performance results

**Fig. 4:**
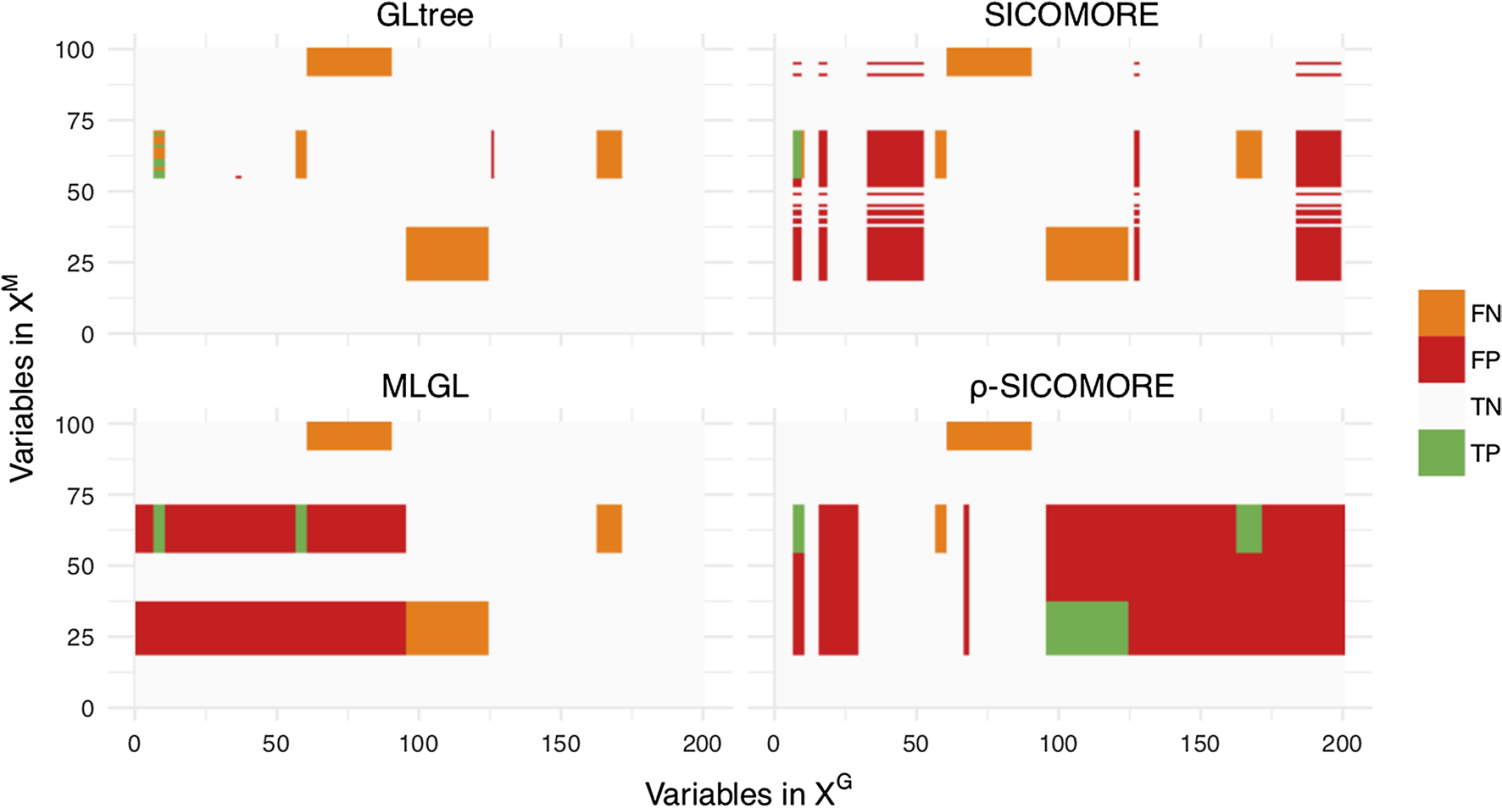
Confusion matrices of interactions $\hat{\theta }_{jj'}$ for the different methods, using the following simulation parameters: $I = 5$, $\sigma = 0.5$, $n=100$. We can see from this example that MLGL and $\rho$-SICOMORE behave similarly, with very large genomic regions identified. SICOMORE tends to work with smaller genomic and metagenomic regions

**Fig. 5:**
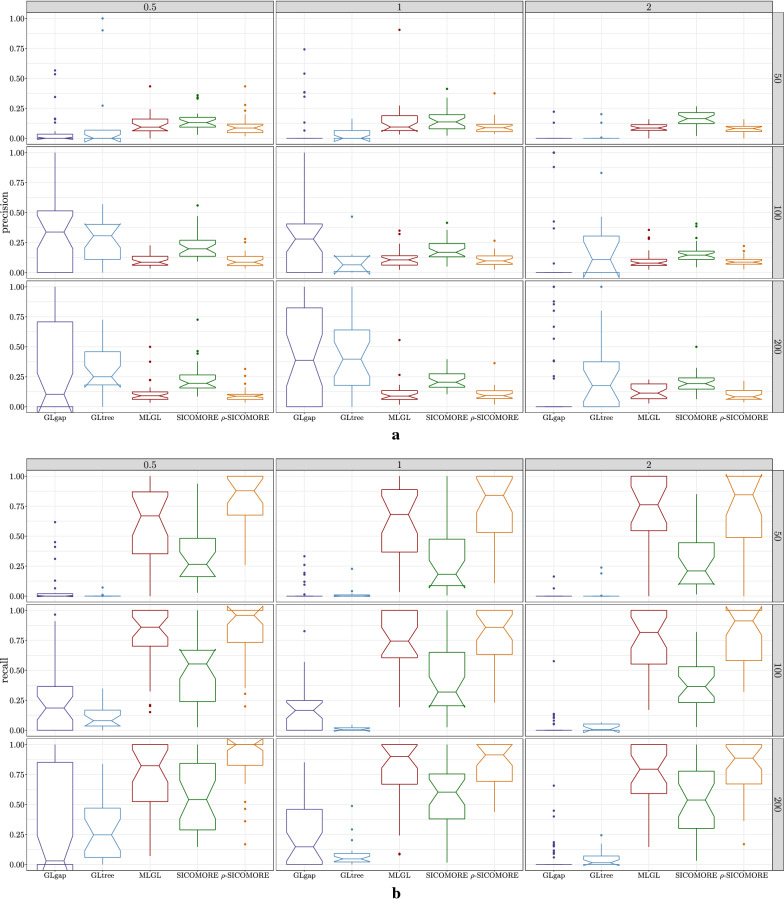
Boxplots of **a** Precision and **b** recall results obtained on the numerical simulations with a Bonferroni-Holm correction for $I=7$ blocks. The lines correspond to different numbers of observations (top: $N=50$, middle: $N=100$ and bottom: $N=200$), and the columns correspond to levels of difficulty of the problem (left: $\epsilon =0.5$, middle: $\epsilon =1$ and right: $\epsilon =2$). The boxplots are best seen in colors: from the left to the right, GLgap is in purple, GLtree is in blue, MLGL is in red, SICOMORE is in green, $\rho$-SICOMORE is in orange

The performances of the different methods in uncovering true causal interactions are shown in Fig. 5a (for Precision) and 5b (for Recall). For the sake of clarity we show only the results for $I=7$ blocks of variables in interaction. The results for $I \in \{1,3,5,10\}$ are provided in Appendix as supplementary results. The plots in Fig. 4 represent the uncovered confusion matrices of interaction $\theta _{gm}$ corresponding to one particular set of simulation parameters ($I = 5$, $\sigma = 0.5$, $n=100$) for each of the compared methods.

The Recall results show that MLGL and $\rho$-SICOMORE are good at uncovering true positive interactions, with $\rho$-SICOMORE performing better overall. SICOMORE performs less well because it favours the selection of small groups that are only partly contained in the groups that generate the interactions. This indicates that MLGL and $\rho$-SICOMORE have an effective weighting scheme. GLgap is unable to uncover relevant interactions, but here the way the structure between variables is defined using the gap statistic differs from the other methods. The Precision results show that all methods perform poorly, with a significant number of false positive interactions. MLGL and $\rho$-SICOMORE tend to select groups of variables and supervariables that are too high in the tree structure, giving rise to false positives that are spatially close to the true interactions. SICOMORE, which, as explained above, favours small groups, gives fewer false positives of this kind. The behaviour of GLgap may vary according to the selected cut with the gap statistic into the tree structure, while the GLtree option has slightly better precision. Note that this improved precision may be the consequence of the additional information provided from our group definition. The glinternet method is mostly unable to uncover the true interactions correctly, whether the compressed or the original representation is used.

### Computation time

In order to reduce the computation time required to run our algorithm, we chose to restrict the search space. It is limited to the area of the tree where the jumps in the hierarchy are the largest, and the number of groups to be evaluated is arbitrarily set to five times the number of initial features. This reduces the number of variables to be fitted in the Lasso regression but does not affect performance regarding Recall and Precision.

We compared the computational performance of our method with the two others by varying the number of variables in $\tilde{\mathbf {X}}^{ G }$. We repeated the number of evaluation five times for each size of $\tilde{\mathbf {X}}^{ G }$ and averaged the computation time.

We can conclude from the results presented in Table 1 that two methods, glinternet and MLGL, are unsuitable for large-scale analyses of genomic data, since computation time starts to rise steeply once the number of variables exceeds a few thousand. The computation time of $\rho$-SICOMORE and SICOMORE is drastically reduced compared to MLGL or glinternet, with $\rho$-SICOMORE having a slight advantage due to the weighting scheme that induces faster elimination of non relevant supervariables.

**Table 1:** Average computation time (in minutes) over 5 replicates for varying dimensions of $\tilde{\mathbf {X}}^{ G }$, with the dimension of $\tilde{\mathbf {X}}^{ G }$ being fixed ($N_{ M }=6$)

$N_{ G }$	50	100	500	1000	1500	2000	3000	4000
$\rho$-SICOMORE	0.01	0.01	0.02	0.03	0.03	0.04	0.05	0.06
SICOMORE	0.21	0.34	0.82	0.76	0.75	0.96	0.93	1.09
MLGL	0.06	0.09	3.35	0.86	3.12	4.52	8.02	24.20
GLtree	0.07	0.28	0.67	3.83	11.69	26.31	88.17	210.64

## Application on the rhizosphere bacterial communities of *Medicago truncatula*

For an implementation of our algorithm on real data we chose to study the interactions between the genome of *Medicago truncatula* and the metagenome (16S rRNA gene sequencing) of its rhizosphere bacterial community. We were seeking to identify significant interactions in order to better understand the effect of both the plant genome and the rhizosphere bacterial microbial community on plant growth.

For this purpose, a core collection of 155 accessions (all from INRAE-Montpellier) were grown in a controlled environment and phenotyped for several traits related to the plant growth and nutritional strategy:

- Total Dry Biomass (TDB).
- Root Total Dry Biomass Ratio (RTDBR).
- Specific Nitrogen Uptake (SNU) expressed as *mg* of $N.g^{-1}$ of belowground biomass per day.

In addition to the phenotypic measurement, the rhizosphere of each accession was also analyzed to determine the bacterial diversity and composition (see Additional file 1). The metabarcoding raw data is available in the European Nucleotide Archive (ENA) EMBL-EBI database system under project accession PRJEB25849.

A total of 15617 different bacterial OTUs were found in the rhizosphere of the plants. The different OTUs were pooled according to their taxonomic affiliation at the genus level, and a total of 329 genera were thus analyzed. The 155 sequenced accessions, extracted from http://www.medicagohapmap.org, were genotyped with a DNA microarray chip, giving a total of 6 372 968 SNPs after 3% MAF, multiallele SNP exclusion and minimum count (100) filtering. The missing values were imputed using the snp.imputation function from the R package snpStats [16]. Given two sets of SNPs typed in the same subjects, this function computes rules that can be used to impute one set from the other in a subsequent sample. By discarding any SNP that had too many missing values to be completely imputed, we reduced the size of the data to 2 148 505 SNPs.

The positions of SNPs inside or in the vicinity of genes (± 2Kb) were extracted from context files downloaded from http://www.medicagohapmap.org. A Singular Enrichment Analysis was conducted using an exact Fisher test with the R package topGO [3] and GO term annotation from http://www.medicagogenome.org.

The algorithm requires several hyper-parameters to be chosen in order to run properly:

- Aggregating function: For the genomic and the metagenomic data, we defined the mean value of the group as supervariable.
- Clustering algorithm: For the metagenomic data we used a hierarchical clustering using Ward’s distance as the measure of similarity. For the genomic data we used a spatially constrained hierarchical clustering algorithm that integrates the linkage disequilibrium as the measure of dissimilarity.
- Stability selection: The parameters of the function stabs in SICOMORE for the metagenomic data were fixed to $\mathtt {B}=300$ subsampling replicates, with the frequency of selection of the supervariables on the replicates $\mathtt {cutoff}=0.7$. The upper bound for the per-family error rate was set to $\mathtt {PFER}=1$. For the genomic data, the parameters were fixed to $\mathtt {B}=100$, $\mathtt {cutoff}=0.6$ and $\mathtt {PFER}=10$.
- Search space: For computational reasons we chose to run some analyses chromosome by chromosome. Correction for multiple testing was done by controlling the false discovery rate [7]. Since weak effects were expected, we also examined interactions with *p*-values $<0.05$ to discuss some aspects in relation with the phenotypes RTDBR and SNU.

Regarding the running time for the application, for about 2M SNPs and 329 bacterial genera, the algorithm was able to perform the analysis in 250 min ($\sim$ 4 hours) with 10 CPU cores (Intel^®^ Xeon^®^ CPU E7-4870 @ 2.40GHz) and 2.5 Gb of memory.

### Results regarding Total Dry Biomass

No significant interactions were found for this phenotype.

### Results regarding the Root Total Dry Biomass Ratio

For RTDBR, four interactions were significant at *p*-value $<0.05$, distributed across three chromosomes, as shown in Table 2. The 365 210 SNPs allow recovering 9 007 genes. A Gene Ontology enrichment analysis carried on the 4 490 annotated genes identified “hormone biosynthetic process” (Fisher *p*-value of $2.10^{-17}$) or “antibiotic biosynthetic process” (Fisher *p*-value of $5.10^{-18}$), “systemic acquired resistance” (Fisher *p*-value of $2.10^{-9}$) and “cellular response to nitrogen starvation” (Fisher *p*-value of $2.10^{-8}$) as four main overrepresented metabolic pathways involved in RTDBR variations under microbe interactions. The three first classes included almost redundant genes, mainly NBS-LRR kinase and 8 transcription factors. The fourth term “cellular response to nitrogen starvation” is composed mainly of lectin-domain receptor kinases genes also present in the three other classes and related to plant defense and of cysteine-rich receptor kinase genes, which are known to be regulated upon biotic and abiotic stress, such as salt and drought stress. For the rhizosphere bacterial communities, 39 genera were found in interaction with these genes. Also, 17, 9, and 6 genera were affiliated to Proteobacteria, Actinobacteria, and Bacteroidtes respectively. Within Proteobacteria, 10 genera were identified as Alphaproteobacteria and 4 of them to the *Rhizobiales* family, which is known to contribute to N nutrition of *Medicago truncatula*. Plant disease resistance genes play a major role in the plant immune system that was induced during pathogenic plant-microbial interactions but also during mutualistic plant-microbe interactions [28].

**Table 2:** Results of the search for interactions using the $\rho$-SICOMORE method

PH	#MG	CHR	GP	#SNPs	*p*-value	*q*-value
RTDBR	39 genera	3	129:980206	6705	0.03	0.18
RTDBR	39 genera	3	980235:32366703	196705	0.04	0.18
RTDBR	39 genera	7	21704918:33495621	68658	0.03	0.23
RTDBR	39 genera	8	50:18024047	93142	0.02	0.14
SNU	180 genera	2	38539843:45729381	33033	0.04	0.13
SNU	180 genera	6	33985403:35275305	6174	0.04	0.13
SNU	180 genera	8	18024755:45569421	156827	0.05	0.09

From left to right, the names of the columns are: PH for the phenotype studied; #MG for the number of genera; CHR for the chromosome; GP for the genomic postion (pb) and #SNPs for the number of SNPs in the genomic region

None of the 39 bacterial genera identified was affiliated to genera known as plant pathogens. However, several of the bacterial genera identified were affiliated to genera known as plant symbiont or plant growth promoting bacteria. We could hypothesize that bacteria affiliated to these genera could be in positive interaction with the plant and induced some defense response.

### Results regarding Specific Nitrogen Uptake

For the SNU, we retrieved 157 698 significant SNPs and 5 476 genes from the three significant interactions, as shown in Table 2. Among the 3 136 annotated genes, the most over-represented biological process was the “transmembrane receptor protein tyrosine kinase signalling pathway” (Fisher *p*-value of $1.10^{-6}$), “regulation of anion channel activity” (Fisher *p*-value of $3.10^{-4}$) and “lignin biosynthesis” (Fisher *p*-value of $8.10^{-4}$).

The two first classes were partly redundant and mainly composed of LRR receptor kinase genes, known to be involved in plant innate immunity. The term “regulation of anion channel activity” was linked to other significant terms related to regulation to ion/anion transport. The “lignin biosynthesis” process included genes involved in lignin biosynthesis such as 8 caffeic acid O-methyltransferase genes, 3 cinnamyl alcohol dehydrogenase-like protein or 2 shikimate O-hydroxycinnamoyltransferase, which serve as building blocks in the formation of plant lignin [63]. The colonization of plant host cells by bacteria involves the progressive remodeling of the plant–microbial interface for both *Rhizobium*-Legume symbiosis [12] and pathogen bacteria [64]. In addition, the plant immune system is involved in symbiosis and during plant pathogen infections, and more generally with the plant microbiota [24, 28]. For the rhizosphere bacterial communities, 180 genera were found in interaction with these genes. 83, 31, 24 and 23 genera were affiliated to Proteobacteria, Firmicutes, Actinobacteria and Bacteroidetes respectively. In addition to the 13 genera belonging to the *Rhizobiales* family, other OTUs were affiliated to bacteria genera harboring functional traits relating to the N cycle, such as nitrogen fixation, nitrate reduction to ammonium, and denitrification, which can contribute to plant nitrogen nutrition.

Altogether, the mathematical method proposed here could support some biological hypothesis that need to be validated using other biological approaches combining plant mutant affected by these genes and simplified bacteria community defined on the genera identified.

## Conclusion

### Synthesis

The detection of interaction effects in a high-dimensional setting remains a difficult problem because multiple testing is onerous and because effects are small in terms of their significance. In this work, we proposed SICOMORE, a method that reduces the dimension of the search space by selecting a subset of compressed variables obtained from the biological characteristics of complementary datasets.

Our approach has demonstrated its ability to uncover interaction effects with a high statistical power. In our simulations, where sample sizes, noise, and the number of true interactions all varied, SICOMORE always exhibited stronger recall than both MLGL and glinternet. SICOMORE combines the strengths of different methods in a powerful single algorithm. SICOMORE is also significantly more efficient than the others in terms of computation time.

SICOMORE was able to detect interactions between the genome of *Medicago truncatula* and its rhizosphere, which are linked to the Root Total Dry Biomass Ratio as well as its Specific Nitrogen Uptake.

### Extensions

Although our approach as presented here concerns the detection of interactions between genomic and metagenomic markers, it should be noted that two major extensions are available.

1. SICOMORE can be applied to any kind of numerical data, as long as an underlying hierarchical or group structure is available (such as a correlation structure, for instance). In particular, our method can handle shotgun sequencing as well as other omics data, or even clinical follow-up, which often takes the form of categorical data that can be easily structured.
2. The compression scheme used in SICOMORE means that the model of interactions can easily be extended to $V > 2$ different datasets. This opens the way to tackling a variety of other problems where different sources of information may be utilized, such as in precision medicine, for instance.

The R package already incorporates these two possibilities.

### Perspectives

Given these interesting results and possible extensions, there are other aspects that may be interesting to address in future works, with a view to improving SICOMORE further in terms of model consistency. Although the Lasso procedure is relevant for dimension reduction purposes, it may induce some biases in the multiple testing procedure used afterwards, since the variable selection step is performed before the *p*-values are adjusted. One way around this problem might be to use post-hoc inference for multiple comparisons [22]. These kinds of extensions should have a positive impact on precision results.

### Supplementary information

**Additional file 1.** Supplemental data for the *Medicago truncatula* example.

## Figures and Tables

**Fig. 6:**
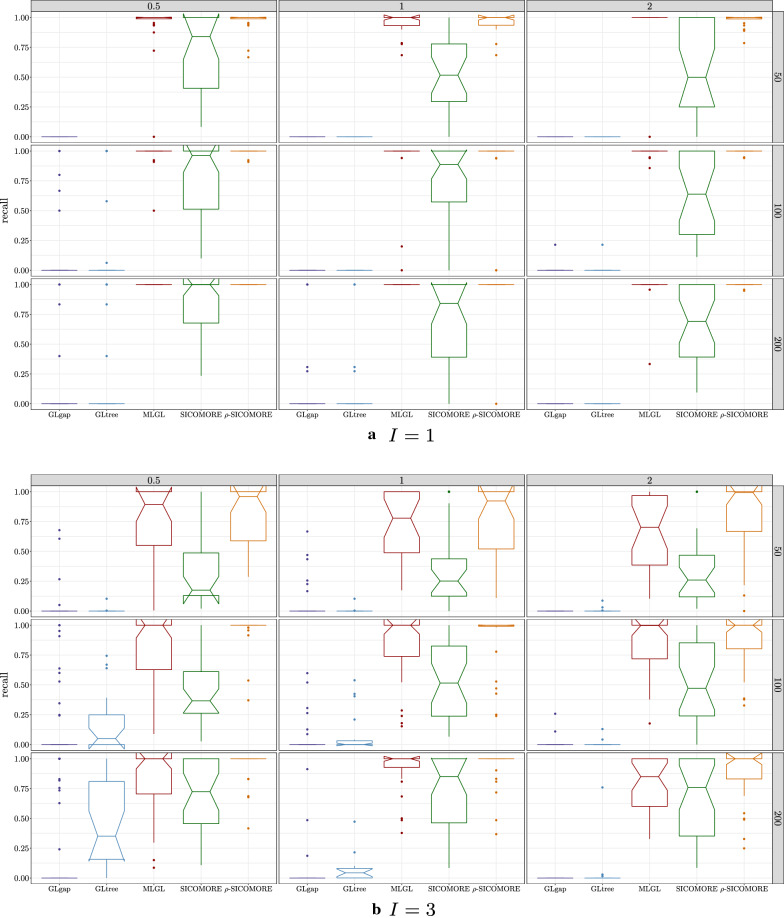

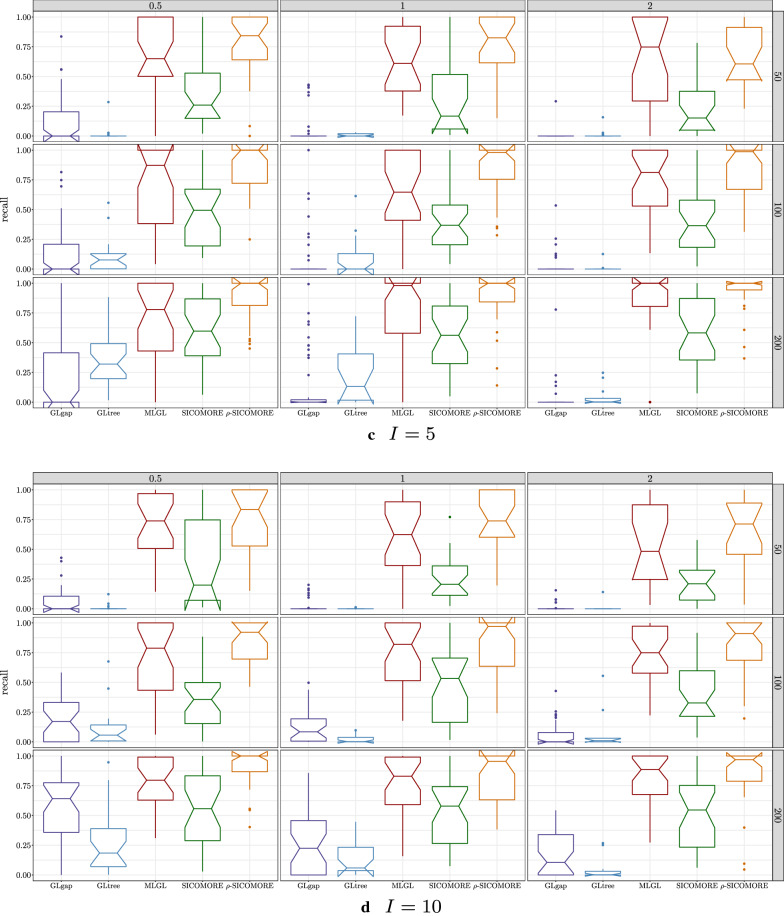
Boxplots for Recall obtained on the numerical simulations with a Bonferroni-Holm correction for *I* = {1, 3, 5, 10} blocks. The lines show the results for different number of observations (top: *N* = 50, middle: *N* = 100 and bottom: *N* = 200) and the columns the difficulty of the problem (left: ε = 0.5, middle: ε = 1 and right: ε = 2). The boxplots are best seen in colors: from the left to the right, GLgap is in purple, GLtree is in blue, MLGL is in red, SICOMORE is in green, ρ-SICOMORE is in orange

**Fig. 7:**
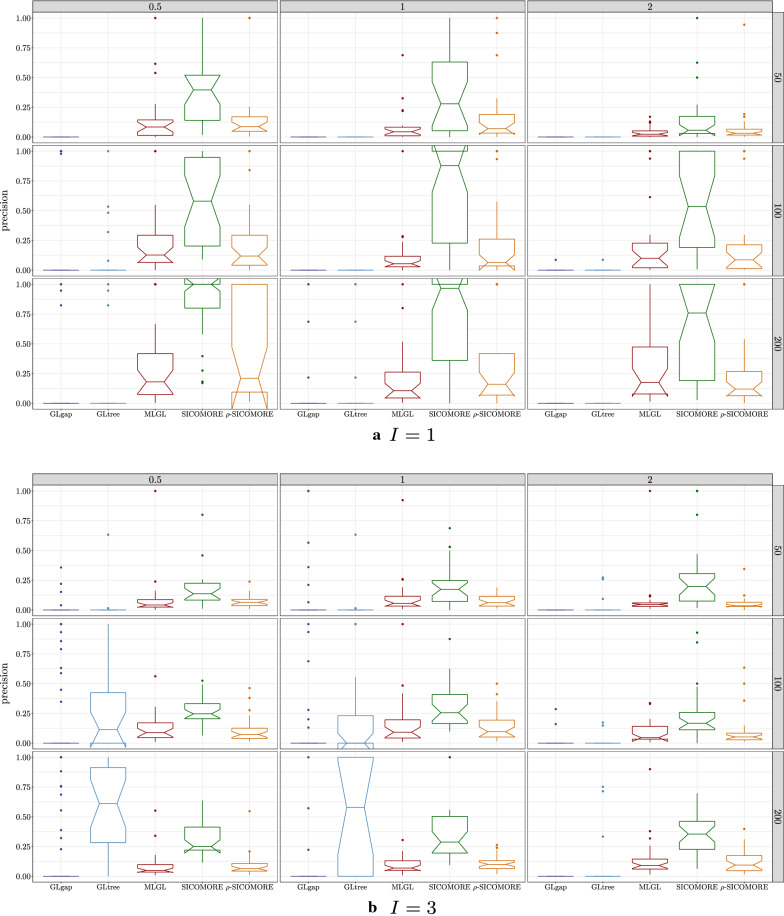

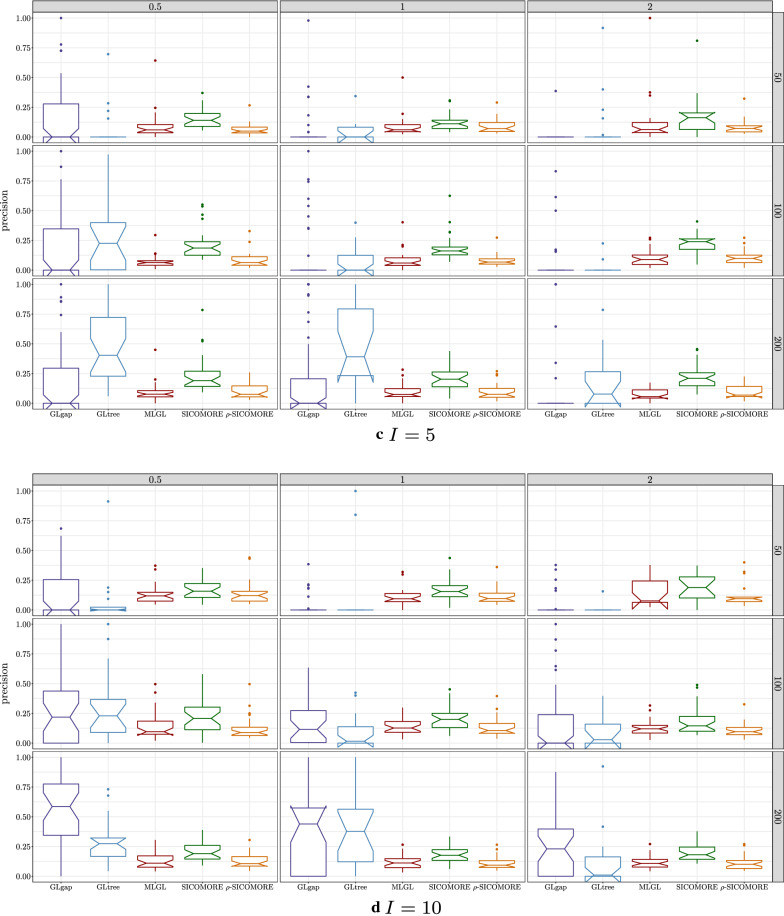
Boxplots for Precision obtained on the numerical simulations with a Bonferroni-Holm correction for *I* = {1, 3, 5, 10} blocks. The lines show the results for different number of observations (top: *N* = 50, middle: *N* = 100 and bottom: *N* = 200) and the columns the difficulty of the problem (left: ε = 0.5, middle: ε = 1 and right: ε = 2). The boxplots are best seen in colors: from the left to the right, GLgap is in purple, GLtree is in blue, MLGL is in red, SICOMORE is in green, ρ-SICOMORE is in orangeíí

## Data Availability

An R package is available, along with its documentation and associated scripts, allowing the reader to reproduce the results presented in the paper [4].

## Appendix

See Figs. 6 and 7.

## Footnotes

1 The rhizosphere was defined by Hiltner in 1904 as the area around a plant root that is inhabited by a unique population of microorganisms influenced by the chemicals released from plant roots.

2 To our knowledge, their implementation based on an alternating direction method of multipliers is not publicly available.

3 http://www.cephb.fr.

4 In GLtree, a matrix $\tilde{\mathbf {X}}$ is created using the initial *D* variables, and the $(D-1)$ groups of variables of the dendogram from the hierarchical clustering are added as compressed features.

## References

[1] Aitchison J, Ho CH (1989). The multivariate poisson-log normal distribution. Biometrika.

[2] Aitchison J (1982). The statistical analysis of compositional data. J Royal Stat Soc Series B.

[3] Alexa A, Rahnenfuhrer J. topGO:Enrichment Analysis for Gene Ontology. (2019). R package version 3.10. 10.18129/B9.bioc.topGO 2019.

[4] Ambroise C, Chiquet J, Guinot F, Szafranski M. sicomore: Selection of Interaction Effects in Compressed Multiple Omics Representations. (2020). R package version 0.2.1. http://julien.cremeriefamily.info/sicomore-pkg/ 2020.

[5] Bach F. Bolasso: model consistent lasso estimation through the bootstrap. In: Proceedings of the 25th Annual International Conference on Machine Learning, 2008;33–40.

[6] Benjamin H, Hothorn T. stabs: Stability Selection with Error Control. (2017). R package version 0.6-3. https://cran.r-project.org/package=stabs 2017.

[7] Benjamini Y, Hochberg Y (1995). Controlling the false discovery rate: a practical and powerful approach to multiple testing. J Royal Stat Soc Series B.

[8] Berendsen RL, Pieterse CMJ, Bakker PAHM (2012). The rhizosphere microbiome and plant health. Trends Plant Sci.

[9] Bergelson J, Mittelstrass J, Horton MW (2019). Characterizing both bacteria and fungi improves understanding of the arabidopsis root microbiome. Sci Rep.

[10] Bien J, Taylor J, Tibshirani R (2013). A Lasso for hierarchical interactions. Annals of statistics.

[11] Blaxter M, Mann J, Chapman T, Thomas F, Whitton C, Floyd R, Abebe E (2005). Defining operational taxonomic units using dna barcode data. Philos Trans Royal Soc B.

[12] Brewin NJ (2004). Plant cell wall remodelling in the rhizobium-legume symbiosis. Crit Rev Plant Sci.

[13] Callahan BJ, McMurdie PJ, Holmes SP (2017). Exact sequence variants should replace operational taxonomic units in marker-gene data analysis. ISME J.

[14] Chevalier J-A, Salmon J, Thirion B. Statistical Inference with Ensemble of Clustered Desparsified Lasso. arXiv:1806.05829 2018.

[15] Clavel J (2007). Progress in the epidemiological understanding of gene-environment interactions in major diseases: cancer. Comptes rendus biologies.

[16] Clayton D. snpStats:SnpMatrix and XSnpMatrix Classes and Methods. (2019). R package version 3.10. 10.18129/B9.bioc.snpStats 2019.

[17] Dehman A, Ambroise C, Neuvial P (2015). Performance of a blockwise approach in variable selection using linkage disequilibrium information. BMC Bioinformatics.

[18] Donoho DL, Tsaig Y (2008). Fast solution of-norm minimization problems when the solution may be sparse. IEEE Trans Inf Theory.

[19] Fischer M, Strauch B, Renard BY (2017). Abundance estimation and differential testing on strain level in metagenomics data. Bioinformatics.

[20] Gloor GB, Macklaim JM, Vu M, Fernandes AD (2016). Compositional uncertainty should not be ignored in high-throughput sequencing data analysis. Austrian J Stat.

[21] Gloor GB, Macklaim JM, Pawlowsky-Glahn V, Egozcue JJ (2017). Microbiome datasets are compositional: and this is not optional. Front Microbiol.

[22] Goeman JJ, Solari A (2011). Multiple testing for exploratory research. Stat Sci.

[23] Gordon AD (1999). Classification. Monographs on statistics and applied probability.

[24] Gourion B, Berrabah F, Ratet P, Stacey G (2015). Rhizobium-legume symbioses: the crucial role of plant immunity. Trends Plant Sci.

[25] Grimonprez Q. Sélection de groupes de variables corrélées en grande dimension. PhD thesis, Université de Lille 2016.

[26] Grimonprez Q, Blanck S, Celisse A, Marot G, Yang Y, Zou H. MLGL: Multi-Layer Group-Lasso. (2020). R package version 0.6-1. https://cran.r-project.org/package=MLGL 2020.

[27] Guinot F, Szafranski M, Ambroise C, Samson F (2018). Learning the optimal scale for GWAS through hierarchical SNP aggregation. BMC Bioinform.

[28] Hacquard S, Spaepen S, Garrido-Oter R, Schulze-Lefert P (2017). Interplay between innate immunity and the plant microbiota. Annual Rev Phytopathol.

[29] Han SS, Chatterjee N (2018). Review of statistical methods for gene-environment interaction analysis. Curr Epidemiol Rep.

[30] Hancock AM, Brachi B, Faure N, Horton MW, Jarymowycz LB, Sperone FG, Toomajian C, Roux F, Bergelson J (2011). Adaptation to climate across the arabidopsis thaliana genome. Science.

[31] Hassani MA, Durán P, Hacquard S (2018). Microbial interactions within the plant holobiont. Microbiome.

[32] Hawe JS, Theis FJ, Heinig M (2019). Inferring interaction networks from multi-comics data-a review. Front Genet.

[33] Hofner B, Boccuto L, Göker M (2015). Controlling false discoveries in high-dimensional situations: boosting with stability selection. BMC Bioinform.

[34] Horton MW, Bodenhausen N, Beilsmith K, Meng D, Muegge BD, Subramanian S, Vetter MM, Vilhjálmsson BJ, Nordborg M, Gordon JI (2014). Genome-wide association study of arabidopsis thaliana leaf microbial community. Nat Commun.

[35] Huang S, Chaudhary K, Garmire LX. More is better: Recent progress in multi-omics data integration methods. Frontiers in Genetics. 2017;8:10.3389/fgene.2017.00084 PMC5472696 28670325

[36] Hutter CM, Mechanic LE, Chatterjee N, Kraft P, Gillanders EM, Tank NG-ET (2013). Gene-environment interactions in cancer epidemiology: a national cancer institute think tank report. Genet Epidemiol.

[37] Jacob L, Obozinski G, Vert J-P. Group Lasso with overlap and graph Lasso. In: Proceedings of the 26th Annual International Conference on Machine Learning, 2009;33–440.

[38] Knights D, Silverberg MS, Weersma RK, Gevers D, Dijkstra G, Huang H, Tyler AD, Van Sommeren S, Imhann F, Stempak JM (2014). Complex host genetics influence the microbiome in inflammatory bowel disease. Genome Med.

[39] Lee S, Görnitz N, Xing EP, Heckerman D, Lippert C. Ensembles of Lasso screening rules. IEEE Transactions on Pattern Analysis and Machine Intelligence. 2017;PP(99):1–1. 10.1109/TPAMI.2017.2765321 PMC6925025 29989981

[40] Li H (2015). Microbiome, metagenomics, and high-dimensional compositional data analysis. Ann Rev Stat Appl.

[41] Li Y, Wu F-X, Ngom A (2016). A review on machine learning principles for multi-view biological data integration. Briefings in Bioinformatics.

[42] Lim M, Hastie T (2015). Learning interactions via hierarchical group-Lasso regularization. J Comput Graph Stat.

[43] Lim M, Hastie T. glinternet: Learning Interactions Via Hierarchical Group-Lasso Regularization. (2019). R package version 1.0.10. https://cran.r-project.org/package=glinternet 2019. 10.1080/10618600.2014.938812 PMC4706754 26759522

[44] Lin X, Lee S, Christiani DC, Lin X (2013). Test for interactions between a genetic marker set and environment in generalized linear models. Biostatistics.

[45] Lugtenberg B, Kamilova F (2009). Plant-growth-promoting rhizobacteria. Annual review of microbiology.

[46] Manolio TA, Collins FS, Cox NJ, Goldstein DB, Hindorff LA, Hunter DJ, McCarthy MI, Ramos EM, Cardon LR, Chakravarti A, Cho JH, Guttmacher AE, Kong A, Kruglyak L, Mardis E, Rotimi CN, Slatkin M, Valle D, Whittemore AS, Boehnke M, Clark AG, Eichler EE, Gibson G, Haines JL, Mackay TFC, McCarroll SA, Visscher PM (2009). Finding the missing heritability of complex diseases. Nature.

[47] Meinshausen N, Bühlmann P (2010). Stability selection. J Royal Stat Soc Series B.

[48] Milligan GW, Cooper MC (1985). An examination of procedures for determining the number of clusters in a data set. Psychometrika.

[49] Mukerji KG, Manoharachary C, Chamola BP (2002). Techniques in mycorrhizal studies.

[50] Park MY, Hastie T, Tibshirani R (2007). Averaged gene expressions for regression. Biostatistics.

[51] Pearson K (1896). Mathematical contributions to the theory of evolution on a form of spurious correlation which may arise when indices are used in the measurement of organs.. Proc Royal Soci London.

[52] Pinton R, Varanini Z, Nannipieri P (2007). The rhizosphere: biochemistry and organic substances at the soil-plant interface.

[53] Qin J, Li Y, Cai Z, Li S, Zhu J, Zhang F, Liang S, Zhang W, Guan Y, Shen D (2012). A metagenome-wide association study of gut microbiota in type 2 diabetes. Nature.

[54] Rau A. Statistical methods and software for the analysis of transcriptomic data. Habilitation à diriger des recherches, Université d’Evry Val d’Essonne 2017.

[55] Segata N, Izard J, Waldron L, Gevers D, Miropolsky L, Garrett WS, Huttenhower C (2011). Metagenomic biomarker discovery and explanation. Genome Biol.

[56] She Y, Wang Z, Jiang H (2016). Group regularized estimation under structural hierarchy. J Am Stat Assoc.

[57] Srinivas G, Möller S, Künzel S, Zillikens D, Baines JF, Ibrahim SM (2013). Genome-wide mapping of gene-microbiota interactions in susceptibility to autoimmune skin blistering. Nat Commun.

[58] Stanislas V, Dalmasso C, Ambroise C (2017). Eigen-epistasis for detecting gene-gene interactions. BMC Bioinform.

[59] Su Z, Marchini J, Donnelly P. HAPGEN: Version 2. (2011a). Version v2.1.2. https://mathgen.stats.ox.ac.uk/genetics_software/hapgen/hapgen2.html 2011.

[60] Su Z, Marchini J, Donnelly P (2011). HAPGEN2: Simulation of multiple disease SNPs. Bioinformatics.

[61] Thomas D (2010). Gene-environment-wide association studies: emerging approaches. Nat Rev Genet.

[62] Tibshirani R, Walther G, Hastie T (2001). Estimating the number of clusters in a data set via the gap statistic.. J Royal Stat Soc Series B.

[63] Tu Y, Rochfort S, Liu Z, Ran Y, Griffith M, Badenhorst P, Louie GV, Bowman ME, Smith KF, Noel JP, Mouradov A, Spangenbergothers G (2010). Functional analyses of caffeic acid o-methyltransferase and cinnamoyl-coa-reductase genes from perennial ryegrass (lolium perenne). Plant Cell.

[64] Underwood W (2012). The plant cell wall: a dynamic barrier against pathogen invasion. Front Plant Sci.

[65] Wang B, Yao M, Lv L, Ling Z, Li L (2017). The human microbiota in health and disease. Engineering.

[66] Wang J, Jia H (2016). Metagenome-wide association studies: fine-mining the microbiome. Nat Rev Microbiol.

[67] Wang J, Thingholm LB, Skiecevičienė J, Rausch P, Kummen M, Kummen M, Hov JR, Degenhardt F, Heinsen FA, Rühlemann MC, Szymczak S (2016). Genome-wide association analysis identifies variation in vitamin D receptor and other host factors influencing the gut microbiota. Nat Genet.

[68] Ward JHJ (1963). Hierarchical grouping to optimize an objective function. J Am Stat Assoc.

