# Supplementary material for: Fast computation of genome-metagenome interaction effects
Source: Algorithms Mol Biol. 2020 Jul 1;15:13. doi: 10.1186/s13015-020-00173-2 (PMC7329492; doi:10.1186/s13015-020-00173-2)
Supplement: Supplementary file 1 — Additional file 1. Supplemental data for the Medicago truncatula example. [file 13015_2020_173_MOESM1_ESM.pdf]

Fast Computation of Genome-Metagenome Interaction Effects

Supplemental data for the *Medicago truncatula* example

With this supplemental data, we intend to provide details to explain the metabarcoding analysis leading to the OTUs used in the *Medicago truncatula* example. This is a part of a side research paper in preparation of Anouk Zancarini, Christine Le Signor and Christophe Mougel.

Metabarcoding analysis

To assess the bacterial communities, the variable region V4 of the 16S rRNA gene was amplified using the 479F and 888R primers and sequenced using Illumina MiSeq sequencing technology (paired-end 2×250 pb). Bioinformatic analyses were done using the GnS-PIPE developed by the GenoSol platform (INRA, Dijon, France) [Terrat et al., 2012]. The details of all steps have been already described previously [Terrat et al., 2015].

After preprocessing, alignment and clustering of reads at 95% of similarity, a filtering step was carried out to check all single-singletons [1] to eliminate PCR chimeras and large sequencing errors produced by the PCR step, based on the quality of their taxonomic assignments. More precisely, each single-singleton was compared with a dedicated reference database from the Silva curated database using similarity approaches (USEARCH), with sequences longer than 500 nucleotides, and kept only if their identity was higher than the defined threshold (95%). The number of high-quality reads for each sample [2] was normalized by random selection to allow an efficient comparison of the datasets and to avoid biased community comparisons.

Then, as the analysis of microbial community richness relies on the construction of similarity clusters (called OTUs), we chose here to use OTUs to examine the distribution of 16S rRNA gene sequences in our datasets. This clustering was realized with a Perl script program that groups rare reads to abundant ones, and does not count the differences in homopolymer lengths. Finally, the global contingency table of OTUs was obtained with the samples in lines and the OTUs in columns, indicating the number of reads in each OTU for all samples. The taxonomy of each OTU was determined based on the taxonomy of all reads encompassed in the OTU. More precisely, an OTU composed of more than 90% of reads of a given taxonomy is assigned to this taxonomy. The community structure was then characterized using weighted UniFrac distance [Lozupone and Knight, 2005] calculated with the PycoGent package [Knight et al., 2007] on a phylogenetic tree computed using FastTree and the most abundant sequence to represent each OTU.

One sample was removed because of its too low-depth [Weiss et al., 2017]. The OTUs with counts lower than 41 over all the samples were filtered. The threshold of 41 was determined using the following procedure: for a threshold varying from 1 to 150, we calculated the number of OTUs whose total counts over all the samples is below this threshold. The selected threshold is the one for which the number of OTUs does not increase when the threshold increases by one. Then, the number of reads in each OTU was first summed for the three replicates for each plant genotype and a between-sample normalization was performed in order to correct for the different sequencing depth. Each sample was scaled by a size factor calculated as the ratio between the total number of counts in this sample and the mean of total counts across all samples. Finally, for each plant genotype, the number of reads were summed for OTU belonging to the same genus. OTUs that had unknown taxonomic assignment at genus level were discarded. Thus, a total of 155 samples and 329 genus were finally analysed.

All raw data sets are publicly available in the European Nucleotide Archive (ENA) of EMBL-EBI database system under project accession PRJEB25849 entitled "*Genome-wide association study of Medicago truncatula rhizosphere microbial communities and plant nutritional strategies*" with raw sequences accession (ERR2495157 to ERR2495714).

Taxonomic affiliations of OTUs

We provide a pie chart that depicts the taxonomic affiliation of the OTUs at phylum level in Figure 1. This results will be presented as a boxplot and discussed in the side paper still in preparation.

[1] Single-singletons are reads detected only once and not clustered.

[2] There are 10 000 high-quality reads for each sample.

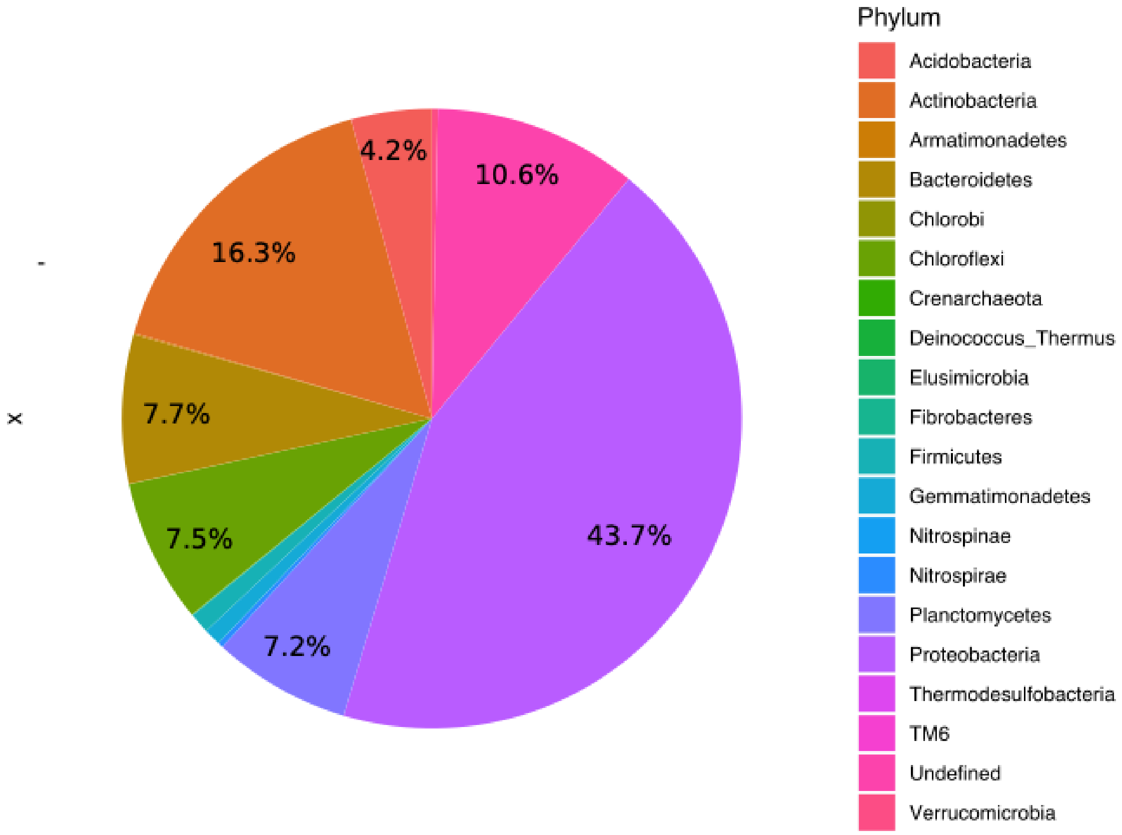

Figure 1: Taxonomic affiliation of the OTUs at phylum level.

References

R. Knight, P. Maxwell, A. Birmingham, J. Carnes, J. G. Caporaso, B. C. Easton, M. Eaton, M. Hamady, H. Lindsay, Z. Liu, et al. Pycogent: a toolkit for making sense from sequence. *Genome biology*, 8(8):R171, 2007.

C. Lozupone and R. Knight. Unifrac: a new phylogenetic method for comparing microbial communities. *Applied and Environmental Microbiology*, 71(12):8228–8235, 2005.

S. Terrat, R. Christen, S. Dequiedt, M. Lelièvre, V. Nowak, T. Regnier, D. Bachar, P. Plassart, P. Wincker, C. Jolivet, A. Bispo, P. Lemanceau, P.-A. Maron, C. Mougel, and L. Ranjard. Molecular biomass and metataxogenomic assessment of soil microbial communities as influenced by soil dna extraction procedure. *Microbial biotechnology*, 5 (1):135–141, 2012.

S. Terrat, P. Plassart, E. Bourgeois, S. Ferreira, S. Dequiedt, N. Adele-Dit-De-Renseville, P. Lemanceau, A. Bispo, A. Chabbi, P.-A. Maron, and L. Ranjard. Meta-barcoded evaluation of the iso standard 11063 dna extraction procedure to characterize soil bacterial and fungal community diversity and composition. *Microbial biotechnology*, 8 (1):131–142, 2015.

S. Weiss, Z. Z. Xu, S. Peddada, A. Amir, K. Bittinger, A. Gonzalez, C. Lozupone, J. R. Zaneveld, Y. Vázquez-Baeza, A. Birmingham, et al. Normalization and microbial differential abundance strategies depend upon data characteristics. *Microbiome*, 5(1):27, 2017.
